# The Yin and the Yang of Transformative Research During the COVID-19 Pandemic—A Perspective

**DOI:** 10.3389/fped.2021.650302

**Published:** 2021-06-24

**Authors:** Venkatesh Sampath, Ramani Ramchandran

**Affiliations:** ^1^Division of Neonatology, Children's Mercy Kansas City, Kansas City, MO, United States; ^2^Department of Pediatrics, University of Missouri at Kansas City, Kansas City, MO, United States; ^3^Department of Pediatrics, Division of Neonatology, Medical College of Wisconsin, Milwaukee, WI, United States

**Keywords:** research, training & development, basic and clinical sciences, transformative team science, COVID-19, rare and complex disease, broad scientific training

## Abstract

The COVID-19 pandemic has highlighted the necessity for scientists from diverse disciplines to collaboratively mitigate the singular calamity facing humanity this century. The ability of researchers to combine exponential advances in technology and scientific acumen has resulted in landmark discoveries in pediatric research and is surmounting the COVID-19 challenge. Several of these discoveries exist in a realm of research that is not classically “basic” or “clinical.” Translational research characterizes this domain partially, but does not fully capture the integrated research approaches that have spurred these discoveries. Herein, we share our perspective on the common themes underpinning the basic and clinical research. We also highlight major differences in the scope, emphasis, approach, and limitations of basic and clinical research that impede multi-disciplinary approaches that facilitate truly transformative research. These differences in research thinking and methodology are ingrained during training wherein the limitations of the chosen discipline, and strengths of alternate disciplines are not adequately explored. Insular approaches are particularly limited in impacting complex diseases pathophysiology in the era of precision medicine. We propose that integration of -omics technologies, systems biology, adaptive clinical trial designs, humanized animal models, and precision pre-clinical model systems must be incorporated into research training of future scientists. Several initiatives from the NIH and other institutions are facilitating such broad-based “research without frontiers” training that paves the way for seamless, multi-disciplinary, research. Such efforts become “transformative” when scientific challenges are tackled in partnership with a willingness to share ideas, tackle challenges, and develop tools/models from the very beginning.

“*The motion of yin and yang generates all things in nature”*

-*Meh Jiuzhang & Guo Lei*

*(A general introduction to Traditional Chinese Medicine, 2010)*

## Introduction

**Figure 1:**
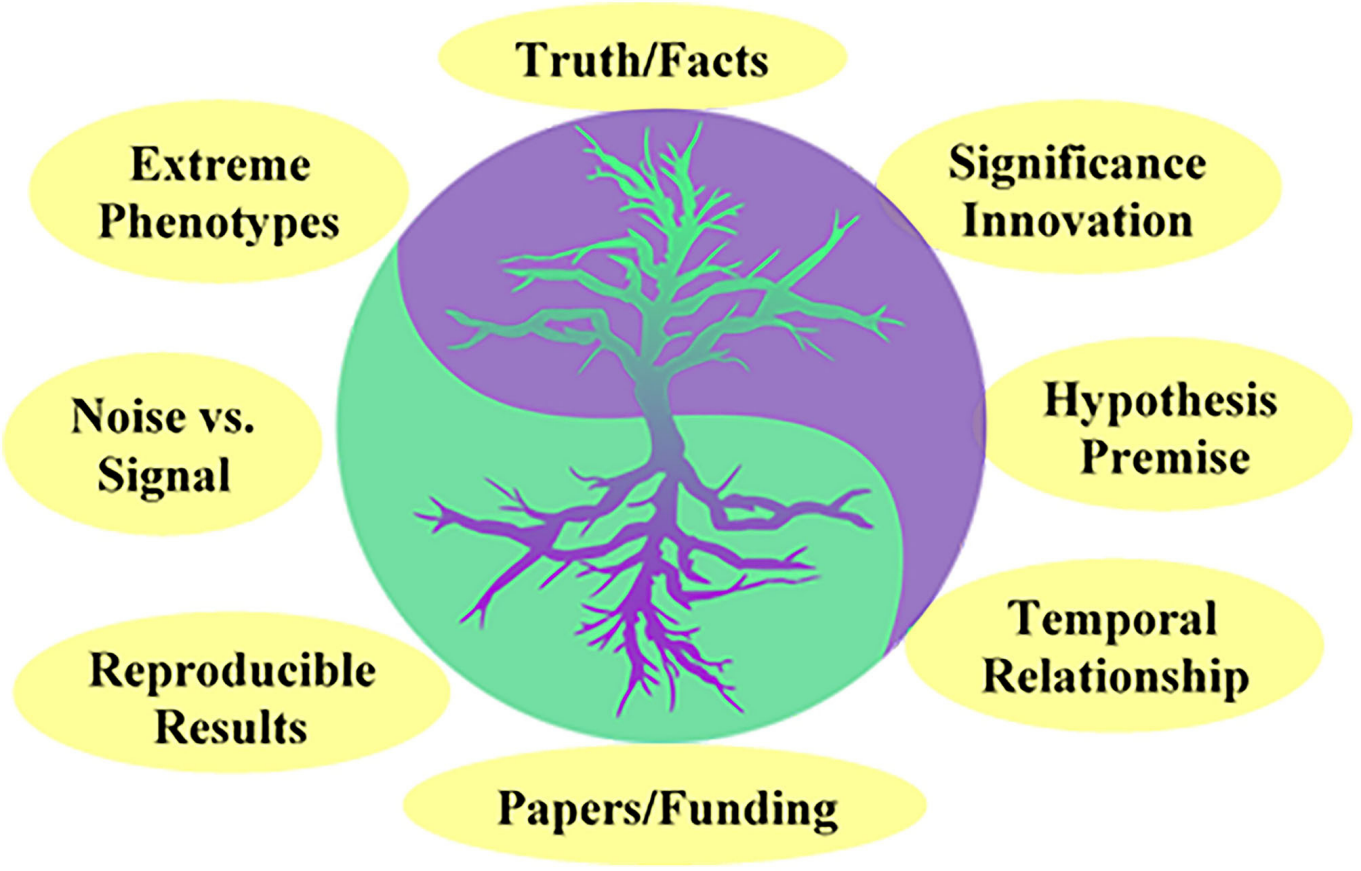
The Yin and the Yang of Transformative research. Commonalities in conception, approach, and goals among clinical and basic scientists. The cartoon suggests that basic and clinical research, respectively, serve as the foundation and support for each other.

The COVID-19 pandemic has highlighted the critical need for clinicians, epidemiologists, basic scientists, and industry to work together to find time-sensitive solutions in a state of chaos. Developing tools for rapid clinical diagnosis/testing, AI-based algorithms to predict disease patterns under different large-scale epidemiological “distancing” measures, characterization of viral pathogenesis, repurposing anti-viral/other therapies, creative bioengineering solutions to personal protective equipment, and innovative clinical trial design is required to react to a rapidly spreading disease (1–3). Historically, and not surprisingly, landmark discoveries in pediatric research such as surfactant treatment in preterm babies, cure of acute lymphoblastic leukemia, lifesaving pediatric vaccinations, and preventing perinatal HIV transmission present outstanding examples of transformative research achieved using a team science approach (4). Basic and clinical scientists represent the yin and the yang of transformative research (5). On the yin side, while the Vannevar Bush report to the US president in 1945 identified that basic scientists focus on discovering the mechanisms that regulate fundamental cellular process or molecular signaling (6), recent interpretations define it as research that provides the foundation of knowledge for applied sciences (7). On the yang side of the research spectrum, clinical scientists focus on discovering factors; epidemiological, exposures, and drugs that impact disease outcomes (8). Traditionally, translational research has been accomplished by basic and clinical scientists working in silos even while building on each other's platform/data independently (Figure 1). Transformative research is accomplished when scientific/medical challenges are tackled as a team from the beginning by basic and clinical scientists working with free exchange of scientific ideas, limitations, strengths, and processes.

## Similarities in Approaches/Perspective Among Basic and Clinical Scientists

Common principles underpin both scientific approaches, and each discipline lays the foundation for the other (Figure 1). Both groups of researchers aim for discoveries that are significant, novel, and have a wide impact, whether it be at a molecular or cellular level or be directly relevant to human disease. The rigorous pursuit of knowledge founded on premise, which facilitates microscopic or macroscopic understanding of disease phenomena is shared. The impetus is on clarifying the mechanism in basic science, while in clinical research it is the impact on human traits, behavior and disease. Once the hypothesis to be investigated is determined, the emphasis on filtering noise and honing on the signal is a critical need for achieving success in both disciplines. A common approach adopted to discern signal from noise is the focus on extreme phenotypes, as with transgenic knock-out mice in basic research, and severe or early onset disease phenotypes for clinical research. Key components of research strategy are also shared, albeit to varying degrees, include consideration of temporal and biological relationships, and reproducibility of results. While common themes pervade basic and clinical research, gravid differences in the conception, conduct, goals, and priorities for each discipline can favor “silo” approaches that limit the transformative collaborative power of the two groups (9).

## Major Differences in Application of the Scientific Process Between Clinical and Basic Scientists

Basic scientists have been trained to investigate mechanisms underpinning cellular processes and molecular function using a step wise hypothesis-driven approach (Table 1). The approach is highly reductionist as it strives to eliminate confounders by controlling the environmental, temporal, and genetic variables. Risk taking in approaches or hypothesis is encouraged as innovation is a key driver for discovery and significance. The burden of proof is often based on confirming Koch's postulates and demonstrating multiple lines of evidence in the model organism. As such, the predominantly linear study design eliminates noise and stochastic behavior to ensure data obtained are valid and reproducible. The major limitations of basic research as it relates to the human phenotypes, especially complex human diseases/traits, is the multi-factorial, chronic, and difficult to model waxing and waning of nature of clinical disease. Experiments in double- or triple-transgenic mice may generate “pure and clean” data but are not reflective of the evolution of human disease *in natura*, that significance is limited. Clinical scientists focus on investigating the effect of exposures, epidemiological, and drugs on human traits or phenotypes using a premise-driven approach. A high risk, high-reward strategy is not feasible, as the potential for harm in subjects is a major consideration. Clinical studies generally use a non-reductionist and inclusive approach, and confounders are not eliminated but rather controlled in the study design with randomization and stratification. The burden of proof lies in statistically confirming or negating the null hypothesis, i.e., that there is no difference between studies groups. The non-linear, non-reductionist design is more likely to capture stochastic behavior, and results of the study are directly relevant to the human condition. However, the major limitation of clinical research is false discovery error arising from confounding, interaction, and mediation (10). Further, clinical studies are very expensive, and often give negative or unclear results, as controlling genetic and epigenomic backgrounds is difficult to surmount.

**Table 1:** Table describing major differences in goals, scope, conception, methodology, and challenges underlying basic and clinical research.

	**Basic**	**Clinical**
Subjects	Animal models, cells	Human subjects
Scope	*Mechanism-centric* Focus on molecular function and interaction. *Microscopic*	*Disease-centric* Focus on a phenotype, disease or human trait. *Macroscopic*
Significance	Study of fundamental biology, physiology, or disease mechanism Direct relevance to human disease not critical	Study of epidemiology, exposures drugs etc., that impact disease Direct relevance to human condition required
Innovation	Focus on discovery technical innovation often high	Focus on effect on phenotype Technical innovation not the focus
Emphasis/ Strategy	Predominantly hypothesis-driven Probes depth of science	Predominantly premise-driven Probes breadth of science
Approach	Reductionist Linear models: Genetic, molecular, noxious exposure manipulation and probing signaling *in vitro* or *in vivo* Powered studies easy *Deterministic hypothesis testing*	Non-Reductionist Planar models: Data collection on risk-factors, exposure, or treatment and correlation with disease/trait Powered analysis challenging *Null hypothesis testing*
Validation and discerning signal vs. noise	Proof–Koch's postulates Additive/subtractive experiments, multiple lines of evidence	Burden of proof–Statistical. Randomization, Stratification, and Repeatability
Limitations	Reductionist models often lead to gaps in translational	Non-mechanistic studies can give rise to false associations
Challenges	Human relevance. Reductionism and eliminating stochastic behavior decreases relevance *in natura*	Expensive. Preventing harm limits high-risk studies. Confounders often results in negative results
Training focus	Molecular and cell biology techniques, biochemistry, systems biology, bioinformatics, animal models	Clinical study and trial design, patient genetics, environment, statistical analysis

## Evolution of Translational Research and Research Training in Translational Researchers

Plotinus (204/5–270 C.E.), the founder of Neoplatonism, quoted that “knowledge, if it does not determine action, is dead to us.” This emphasis on actionable scientific knowledge birthed translational science, which has emerged as its own discipline this century. While the central idea of knowledge that is relevant to human disease or health outcomes is established, it has diverse connotations to various scientists (11, 12). To a basic scientist, translational research implies discovery of mechanisms or fundamental biology that can inform clinical studies or trials that may impact health outcomes. From the perspective of a clinical scientist, translational research implies use of data from human studies and clinical trials to enhance our understanding of human disease and improve health outcomes. Currently, broader definitions combine discovery-based science, clinical trials, and implementation science, with a seamless transition from fundamental discovery to clinical applications that advance science relating to human health and disease (11).

Translational research now encompasses a broader vision, and is frequently categorized in 4 phases (T1, T2, T3, and T4) (13, 14). The T1 phase describes the development of basic science discoveries through early phase I clinical trials. T2 phase seeks to establish the efficacy in humans and determining/establishing clinical guidelines. T3 phase involves the implementation and dissemination of phase T2 research results. T4 phase assesses the community impact and effectiveness of clinical interventions. Basic scientists clearly contribute to T1 phase research. We speculate that inclusion of basic scientists in all 4 phases offers better integration, and develops a re-iterative process that increases successful implementation. For example, basic scientist's knowledge of the biological targets and its effect on various pathway *apriori* may inform which populations are likely to see benefits and which are likely to see little benefits or side effects. Similarly, involvement of clinical scientists early in T1 phase, may guide basic scientists in designing experiments and noting pre-clinical end points that are of clinical significance. For example, a clinician's nuanced perspective with relation to human disease will help refine the design of the basic science model. Such early and often interactions also serves the purpose of revealing limitations within each branch of science, which may diffuse misperceptions of individual disciplines, and facilitate a culture shift in how research is designed and conducted. Thankfully, several such initiatives are already underway. For example, the KL2 mentored clinical research scholar award through Clinical and Translational Science Institutes around the country, MD/PhD physician scientist program, MD/MPH, MD/MBA and programs offering a PhD in translational sciences (15–18). At the grassroot training level, more medical students are now gaining short-term research training experiences (T35 fellowships) in M1–M2 years, with an opportunity to continue research training in during their subsequent medical training. Even research opportunities are available during residency training such as the NIH funded physician scientist immersion pathway (R38 funding). These initiatives are a step in the right direction, and with more “translational-minded,” trained workforce emerges in the MD and PhD professions, distinctions between T1 and T4 translational research categories will begin to blur, and the benefits of this interactive training will hopefully yield to productive solutions in healthcare over the next 5–10 years.

## Transformational Research Training in the ERA of Precision Medicine and Complex Phenotypes

Traditional “silo” research training is particularly challenged in the era of complex disease phenotypes and precision medicine. Recognition of the impact of host-dependent variables to heterogeneity in human traits, diseases, and drug responses, mandates adequate training in the role of biological variability in clinical and basic research (15, 19, 20). In the clinical research domain, rigorous consideration of the genetic/epigenetic background, environmental exposures, diet, microbiome, and sex among other factors is often required. However, adequate consideration of these complex variables challenges the economics and conduct of traditional randomized clinical trials (21, 22). Therefore, training in adaptive clinical research designs, which allow accumulating results of a trial to alter its course or end-points in accordance with pre-specified rules, provide an attractive option. Adaptive design trials can be more efficient, informative, and ethical than traditional fixed design, and often make better use of resources such as time, money, and patients (21–23). On the basic science research realm, critics have questioned whether animal models accurately recapitulate human disease pathogenesis or its complexity. However, animal models are critical for *in vivo* causation confirmation of human pathological variants, for understanding disease pathophysiology and critical developmental mechanisms, screening drug testing, and are therefore important in the era of personalized medicine (24, 25). Basic science research training that encompasses careful selection of human diseases that allow animal modeling, use of functional and comparative genomics to build models of human diseases, and the use of personalized models for drug testing and biomarker discovery offer a foundation for transformational research (24–26). Biomarker discovery or validation should incorporate input from basic and clinical scientists, as understanding biology of the molecule, it's temporal kinetics, and its relation to disease pathogenesis and disease-stage (early vs. late), are required to develop robust biomarkers (27). Several initiatives including the NIH-FDA BEST (Biomarkers, EndPoint, and Other Tools) initiative aims to be a “living resource” for biomarkers capturing definitions, hierarchical and temporal relationships to clinical conditions, will benefit from collaborations between basic and clinical scientists (27, 28). Development of the International Mouse Phenotyping Consortium (IMPC), use of humanized mouse models of clinical disease and mouse systems genetics-based approaches are exciting developments that bring basic research to the era of complex diseases and precision medicine (26, 29, 30). Thus, training in “modern” clinical and basic research approaches that embrace biological variability, complex disease evolution and precision medicine will decrease gaps in research translation (9, 15, 31). Such training is likely to result in transformative research as clinical and basic scientists feel less constrained about working together from the beginning to exchange ideas and solutions to medical/research challenges.

## Training Focusing on Rare Diseases

On the other end of the spectrum from complex diseases are rare diseases, which present a unique set of challenges. From the basic science side, lack of ready availability of animal models to study rare phenotypes is an issue, and also the availability of sample materials for research is an issue given the rare condition. Training in CRISPR-Cas9 technologies, that enable rapid generation of specific animal models that genocopy human mutations linked with rare disease is essential. Familiarity with the use of “organ on chip” approaches, novel model systems and macro- and micro-phenotypes of known mouse mutants (IMPC) can bolster traditional basic science approaches (30, 32–34). On the clinical side also, rare disease present a great challenge in terms of recruiting and adequate N for clinical trials. Critical is the formation of international consortia/registry that quickly link patients with disease to available clinical trials. Training in adaptive designs with non-dichotomous outcomes and Bayesian analysis framework is a priority for the future conduct of rare disease research (34–36).

## Conclusion

The turn of the twenty-first century has witnessed an explosion of scientific knowledge in several disciplines but has also highlighted the need for novel research approaches. With the availability of several tools and the emphasis on complex disease and precision medicine, it has become necessary for basic and clinical scientists to collaborate as no laboratory has all the technologies or scientific acumen individually. Importantly, such collaboration is required to develop integrated “research without frontiers” team science approaches that drive transformative research (15, 20, 37). This is happening in real-time with the COVID-19 crisis with close collaborations between diverse scientists, development of tools for rapid testing, repurposing of drugs supported by NIH and pharmaceutical companies, adaptive clinical study designs, AI-based modeling of disease-spread under different scenarios, and potential pre-clinical/*in vitro* models for understanding mechanisms and developing new therapies (1–3). Thus, the COVID-19 “*in natura*” phenomenon may serve as an example of efficiencies, inefficiencies and gaps in transformative research when applied to pandemics or other research challenges. As in nature, combining the yin and the yang of scientific disciplines will unleash the Qi (vital force) required for transformative research.

## Data Availability Statement

The original contributions presented in the study are included in the article/supplementary material, further inquiries can be directed to the corresponding author/s.

## Author Contributions

VS: overall concept and design, drafting the initial manuscript, and figure design. RR: overall concept and design and editing the final manuscript. All authors contributed to the article and approved the submitted version.

## Conflict of Interest

The authors declare that the research was conducted in the absence of any commercial or financial relationships that could be construed as a potential conflict of interest.
